# Effects of Ration Levels on Growth and Reproduction from Larvae to First-Time Spawning in the Female *Gambusia affinis*

**DOI:** 10.3390/ijms16035604

**Published:** 2015-03-11

**Authors:** Zhiming Zhu, Xiangling Zeng, Xiaotao Lin, Zhongneng Xu, Jun Sun

**Affiliations:** Institute of Hydrobiology, Jinan University, Key Laboratory of Aquatic Eutrophication and Control of Harmful Algal Blooms of Guangdong Higher Education Institutes, 601 Huangpu Avenue West, Guangzhou 510632, China; E-Mails: caoxie-zzm@163.com (Z.Z.); zengxiangling620@126.com (X.Z.); txuzn@jnu.edu.cn (Z.X.); tsunjun@jnu.edu.cn (J.S.)

**Keywords:** *Gambusia affinis*, ration levels, somatic growth, gonad development, reproduction, energy allocation

## Abstract

Somatic growth and reproduction were examined in individual laboratory-grown female *Gambusia affinis* fed with high (H), medium (M) and low (L) ration levels from birth to the first-time spawning. Results showed that the body length and weight, condition factor (*CF*), wet weight gain (*WG*_w_), specific growth rate in wet weight (*SGR*_w_) and ration levels in terms of energy (*RL*_e_) decreased significantly (*p* < 0.05) with decreasing ration levels from birth to first-time spawning. On the contrary, the food conversion efficiency in terms of energy (*FCE*_e_) increased significantly (*p* < 0.05) with the decreasing ration levels from birth to first-time sexual maturity. Furthermore, higher percentages of energy intake from food were allocated to somatic and gonad growth in M and L groups compared to the H group before sexual maturity; In addition, the time for first-time spawning in groups M and L was longer than that of the H group. As a result, the gonad-somatic index (*GSI*) and oocytes/embryos weight in M and L groups were similar to that of the H group, although the ovary weight and oocytes/embryos numbers were all lower than that of the H group. Also, similar growth performances were observed in second-generation offspring, which were produced by female parents fed with different ration levels. These findings suggest that the female *G. affinis* could produce a number of healthy offspring under conditions of food restriction, and that this could be achieved by increasing the energy allocated to gonad development, reducing fecundity and delaying spawning time. These life strategies ensured that *G. affinis* could survive and thrive in adverse environmental conditions and exhibit characteristics of invasive fish species.

## 1. Introduction

Resource availability exerts a profound influence on the growth of fish and may affect the reproduction and energy allocation in some fish species [1,2,3]. Characteristics such as body size, gonad-somatic index, fecundity, and the size of the offspring are most dramatically affected by ration levels during reproductive cycles [4,5,6,7].

The western mosquitofish, *Gambusia affinis*, is a small ovoviviparous fish species of the family Poeciliidae that is native to the southern and eastern United States [8,9]. *G. affinis* has been widely introduced for the purpose of mosquito control [9,10]; unfortunately, it has not always been effective in achieving this goal [11,12,13]. In many areas, *G. affinis* has posed a substantial threat to native aquatic species due to the high rate of population growth and aggressive feeding behaviors [8,14,15]. To further enhance the ecological invasion, *G. affinis* has demonstrated the capacity to live and thrive in a wide variety of habitats, and this is partly due to phenotypic plasticity in life history traits such as age and size at maturity and reproductive allocation based on environmental conditions [16,17,18]. It has been reported that food ration levels can influence growth, reproduction, and energy allocation in *G. affini*s during its juvenile and adult stages [19,20,21,22]. These were only preliminary observations, and no subsequent detailed research studies had been conducted to confirm the impact of ration levels on the physiological and reproductive strategies of *G. affinis* to date.

The effects of ration levels on somatic growth, gonad development, reproduction, and energy allocation were examined in female *G. affinis* from birth to first-time reproduction to quantify its phenotypic response to variation in food resource availability. Additionally, the growth of second-generation offspring were also examined to determine the difference in maternal effects in females given different rations. These experimental results will demonstrate the physiology strategies used by female *G. affinis* to adapt its somatic growth and gonad development patterns during different developmental stages to fit the varying energy intake, as well as the adaptation of energy strategies in response to changing environmental conditions. The results also provide valuable information on the specific adaptive mechanism associated with *G. affinis* invasion.

## 2. Results

### 2.1. Growth and Feed Utilization

The survival rates were 98%–100% among experimental groups throughout the experiment period. The body length and weight decreased significantly (*p* < 0.05) with the decreasing ration levels within the three developmental stages (Table 1). No statistical difference (*p* > 0.05) was observed in the *CF* between the M and L groups, however, they were all significantly lower (*p* < 0.05) than the H group within three developmental stages. The *WG*_w_ was decreased significantly (*p* < 0.05) with decreasing ration levels within the three developmental stages. Similarly, the *SGR*_w_ was also decreased significantly (*p* < 0.05) with decreasing ration levels in the “0–16 days of age” stage. No statistical difference (*p* > 0.05) in the *SGR*_w_ was observed between the L and M groups in the other stages, but they were all markedly lower (*p* < 0.05) than the H group. Similarly, a significant decrease (*p* < 0.05) of *RL*_e_ was observed with the decreasing ration levels within the three developmental stages. On the contrary, the *FCE*_e_ was increased significantly (*p* < 0.05) with the decreasing ration levels in the stages of “0–16 days of age” and “17 days of age—First-time sexual maturity”. The negative values of *FCE*_e_ observed in L and M groups, which were all significantly lower (*p* < 0.05) than that of the H group at the “First-time sexual maturity—First-time spawning” stage were surprising.

### 2.2. Energy Allocation

The total energy intake from food decreased with the decreasing ration levels in the stages of “17 days of age—First-time sexual maturity” and “First-time sexual maturity—First-time spawning”. The value in the L group was significantly lower than that of the M group while the value in the M group was also significantly lower than that of the H group (*p* < 0.05) (Table 2). A similar result was obtained for the average energy intake. The absolute energy used for somatic and gonadal growth were decreased significantly (*p* < 0.05) in the L group compared with that in the H and M groups in the stage of “17 days of age—First-time sexual maturity”, and similar results were also observed in the stage of “First-time sexual maturity—First-time spawning”. However, the percentage energy cost of the somatic growth, accounting for the total energy intake, was increased significantly (*p* < 0.05) with decreasing ration levels in the stage “17 days of age—First-time sexual maturity”, and an opposite result was observed in the stage of “First-time sexual maturity—First-time spawning”. Furthermore, the energy cost of gonadal growth in the M and L groups also increased significantly (*p* < 0.05) compared with the H group in the “17 days of age—First-time sexual maturity” stage, but no marked difference (*p* > 0.05) was observed between the M and L groups. However, there was no significant difference (*p* > 0.05) observed in the energy cost of gonadal growth in the three ration levels for the stage of “First-time sexual maturity—First-time spawning”.

### 2.3. Reproduction Parameters

**Figure 1:**
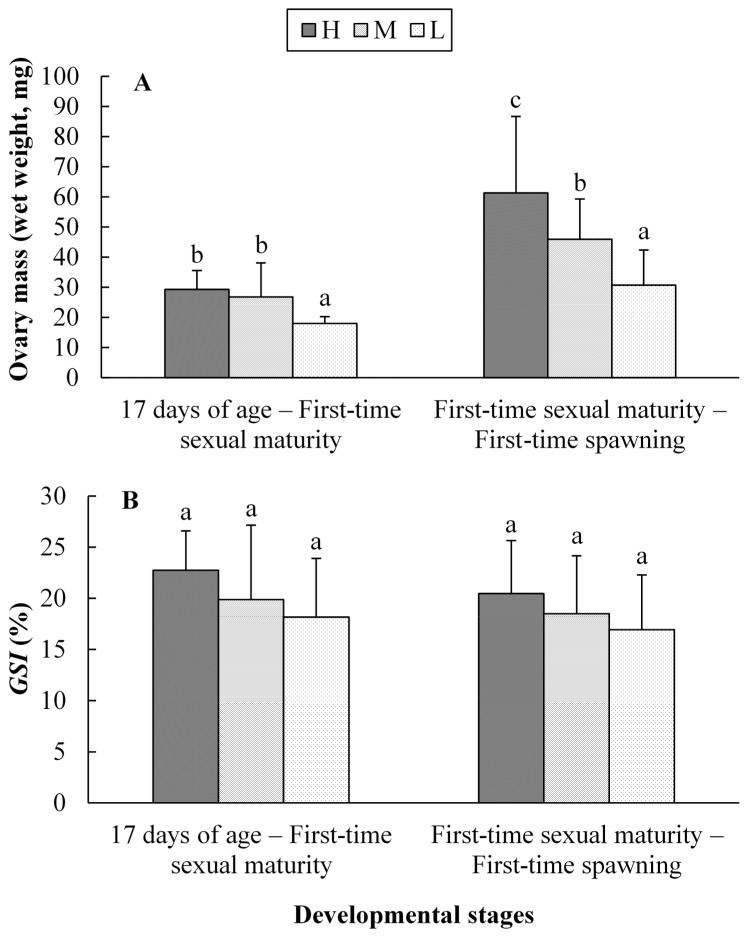
The ovary weight (**A**) and gonad-somatic index (*GSI*) (**B**) of female *G. affinis* at the first-time reproduction for three ration levels during different developmental stages. H: high ration level; M: medium ration level; L: low ration level. Different superscripts within the same developmental stage represent significant differences (one-way ANOVA, *p* < 0.05).

The ovary weight was decreased with the decreasing ration levels both in the stages of “17 days of age—First-time sexual maturity” and “First-time sexual maturity—First-time spawning” (Figure 1). At the stage of “17 days of age—First-time sexual maturity”, the ovary weight in L ration level (average 18.03 mg) was significantly lower (*p* < 0.05) than that in H and M ration levels (average 29.36 and 26.78 mg, respectively). There was no marked difference observed between the H and M groups. At the stage of “First-time sexual maturity—First-time spawning”, the ovary weight in L ration level (average 30.72 mg) was significantly lower (*p* < 0.05) than that in the M ration level (average 45.96 mg). And the ovary weight in the M ration level was also significantly lower (*p* < 0.05) than that in H ration level (average 61.31 mg). However, no marked difference (*p* > 0.05) existed in the *GSI* among the three ration levels in the two developmental stages.

**Table 1:** The growth performances and *FCE*_e_ of fish in three ration levels from birth to first-time spawning.

Developmental Stages	Ration Levels	Body Length (mm)	*n*	Body Weight (Wet Weight, mg)	*CF*	*WG*_w_ (mg·day^−1^)	*SGR*_w_ (%·day^−1^)	*RL*_e_ (%)	*FCE*_e_ (%)
0–16 days of age	H	15.86 ± 1.02 ^c^	40	72.53 ± 14.61 ^c^	1.80 ± 0.20 ^b^	4.01 ± 0.86 ^c^	16.31 ± 1.36 ^c^	34.50 ± 8.68 ^c^	31.68 ± 6.51 ^a^
	M	15.34 ± 0.85 ^b^	40	61.42 ± 7.29 ^b^	1.71 ± 0.18 ^a^	3.35 ± 0.43 ^b^	15.43 ± 0.70 ^b^	22.23 ± 2.34 ^b^	46.87 ± 5.50 ^b^
	L	13.72 ± 0.65 ^a^	40	43.77 ± 5.35 ^a^	1.69 ± 0.12 ^a^	2.31 ± 0.31 ^a^	13.42 ± 0.87 ^a^	13.97 ± 2.06 ^a^	72.05 ± 8.99 ^c^
17 days of age—First-time sexual maturity	H	21.91 ± 1.08 ^c^	60	210.94 ± 27.51 ^b^	2.00 ± 0.18 ^b^	7.29 ± 1.45 ^c^	5.57 ± 0.71 ^b^	119.44 ± 5.14 ^c^	13.96 ± 3.62 ^a^
	M	22.51 ± 0.75 ^b^	60	203.85 ± 25.29 ^b^	1.78 ± 0.15 ^a^	6.19 ± 1.10 ^b^	5.18 ± 0.53 ^a^	98.50 ± 4.03 ^b^	26.23 ± 4.19 ^b^
	L	21.09 ± 0.85 ^a^	60	164.49 ± 15.68 ^a^	1.75 ± 0.12 ^a^	4.64 ± 0.60 ^a^	5.07 ± 0.36 ^a^	57.79 ± 1.29 ^a^	29.59 ± 3.89 ^c^
First-time sexual maturity—First-time spawning	H	23.57 ± 1.51 ^b^	100	272.71 ± 55.73 ^c^	2.06 ± 0.25 ^b^	5.33 ± 2.74 ^c^	1.15 ± 1.09 ^b^	42.45 ± 1.06 ^c^	4.95 ± 5.82 ^b^
	M	23.14 ± 0.78 ^b^	100	236.27 ± 29.62 ^b^	1.91 ± 0.21 ^a^	3.39 ± 1.40 ^b^	0.66 ± 0.60 ^a^	22.55 ± 0.32 ^b^	−0.23 ± 6.23 ^a^
	L	21.48 ± 0.62 ^a^	100	182.47 ± 23.24 ^a^	1.84 ± 0.23 ^a^	0.71 ± 0.92 ^a^	0.38 ± 0.51 ^a^	16.11 ± 0.16 ^a^	−1.71 ± 6.12 ^a^

*FCE*_e_: food conversion efficiency in terms of energy; *CF*: condition factor; *WG*_w_: wet weight gain; *SGR*_w_: specific growth rate in wet weight; *RL*_e_: ration levels in terms of energy; H: high ration level; M: medium ration level; L: low ration level. The different superscripts in each column within the same developmental stage present significant difference (one-way ANOVA, *p* < 0.05).

**Table 2:** Energy allocation of female *G. affinis* at three ration levels in different developmental stages.

Developmental Stages	Ration Levels	*n*	Food Consumption		Energy Allocation (kJ·fish^−^^1^)		As a Percentage of Consumption (%)	
			Total (kJ·fish^−1^)	Average (kJ·g^−1^·day^−1^)	Somatic Growth	Gonadal Growth	Somatic Growth	Gonadal Growth
17 days of age—First-time sexual maturity	H	60	6.81 ± 0.15 ^c^	2.53 ± 0.05 ^c^	0.63 ± 0.01 ^b^	0.40 ± 0.01 ^b^	9.18 ± 0.35 ^a^	5.80 ± 0.16 ^a^
	M	60	4.39 ± 0.06 ^b^	1.44 ± 0.02 ^b^	0.68 ± 0.01 ^b^	0.36 ± 0.00 ^b^	15.56 ± 0.08 ^b^	8.26 ± 0.02 ^b^
	L	60	2.55 ± 0.00 ^a^	0.95 ± 0.00 ^a^	0.53 ± 0.00 ^a^	0.22 ± 0.00 ^a^	20.97 ± 0.33 ^c^	8.63 ± 0.13 ^b^
First-time sexual maturity—First-time spawning	H	100	6.71 ± 0.10 ^c^	1.34 ± 0.00 ^c^	0.23 ± 0.00 ^c^	0.15 ± 0.00 ^b^	3.48 ± 0.42 ^c^	2.24 ± 0.24 ^a^
	M	100	3.70 ± 0.04 ^b^	0.80 ± 0.01 ^b^	0.05 ± 0.00 ^b^	0.06 ± 0.00 ^a^	1.31 ± 0.36 ^b^	1.53 ± 0.76 ^a^
	L	100	2.23 ± 0.00 ^a^	0.51 ± 0.00 ^a^	−0.02 ± 0.00 ^a^	0.06 ± 0.00 ^a^	−0.97 ± 0.40 ^a^	2.74 ± 0.27 ^a^

The different superscripts in each column within the same developmental stage present significant difference (one-way ANOVA, *p* < 0.05).

**Figure 2:**
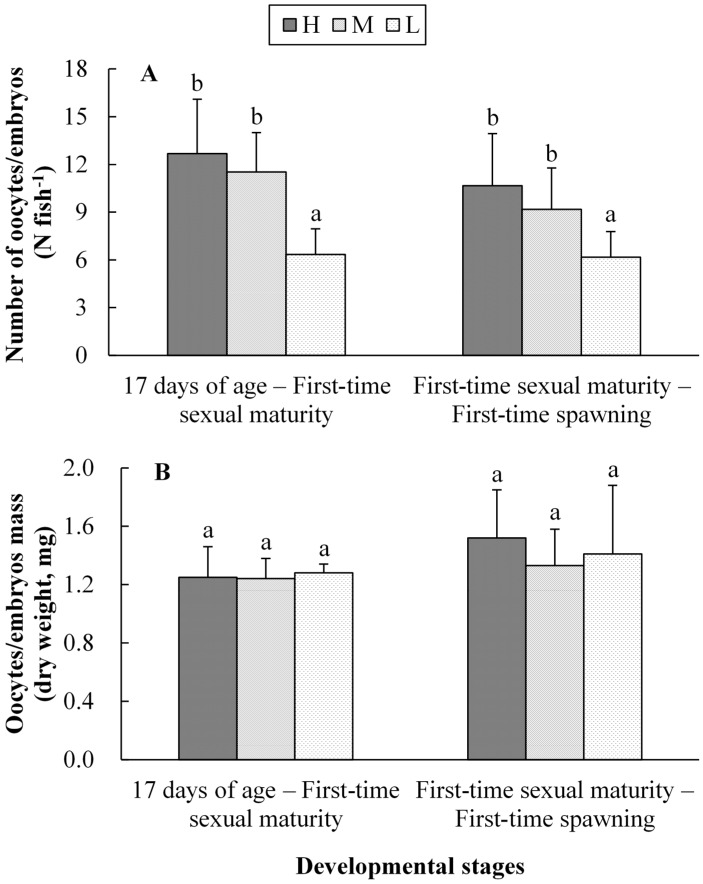
The number (**A**) and weight (**B**) of oocytes (for 17 days of age—First-time sexual maturity stage) or embryos (for First-time sexual maturity—First-time spawning stage) for female *G. affinis* at first-time reproduction in the three ration levels during different developmental stages. Different superscripts within the same developmental stage represent significant difference (one-way ANOVA, *p* < 0.05).

The number of oocytes in H and M ration levels were 12.69 and 11.52 in the stage of “17 days of age—First-time sexual maturity” respectively, and no marked difference (*p* > 0.05) existed between them (Figure 2). However, this number in the L group was 6.35, which was reduced significantly (*p* < 0.05) by 49.96% and 44.88% compared to H and M groups. The times taken to first-time spawning were 52, 55 and 58 days in H, M and L ration levels, respectively (unlisted). The number of embryos in the H, M and L ration levels were 10.66, 9.18 and 6.17, respectively. The number of embryos in the L group decreased significantly (*p* < 0.05) by 42.12% and 32.79% at the stage of “First-time sexual maturity—First-time spawning” when compared with the H and M groups. However, there was no significant difference (*p* > 0.05) observed in the oocytes/embryos weight among the three ration levels in the other two developmental stages.

Table 3 showed the growth performances of the second-generation offspring from 0 to 11 days of age. There was no significant difference observed in the body length, body weight, and *WG*_w_ of the male and female larvae no matter the ration level (H, M or L) fed to their female parents.

**Table 3:** The growth performances of the second-generation offspring from 0 to 11 days of age.

Sex	Ration Levels ^1^	*n*	Body Length (mm)	Body Weight (mg)	*WG*_w_ (mg·day^−1^)
Male	H	10	11.37 ± 0.36 ^a^	21.13 ± 2.46 ^a^	1.27 ± 0.17 ^a^
	M	10	10.98 ± 0.77 ^a^	20.08 ± 3.73 ^a^	1.22 ± 0.12 ^a^
	L	10	11.23 ± 0.70 ^a^	21.20 ± 3.55 ^a^	1.38 ± 0.18 ^a^
Female	H	10	11.52 ± 0.40 ^a^	20.78 ± 2.17 ^a^	1.30 ± 0.19 ^a^
	M	10	11.27 ± 0.43 ^a^	20.52 ± 1.58 ^a^	1.19 ± 0.29 ^a^
	L	10	11.43 ± 0.46 ^a^	22.38 ± 2.29 ^a^	1.29 ± 0.27 ^a^

^1^ means the ration levels of their female parents; different superscripts in each column within the same sex larvae represent significant difference (one-way ANOVA, *p* < 0.05).

## 3. Discussion

We investigated the effects of ration levels on the growth performances and feed utilization of female *G. affinis* from birth to first-time reproduction. In the present study, the high survival rate of female *G. affinis* suggested that the ration levels did not have a devastating effect on them. The food restriction in M and L ration groups led to the significantly decreased *RL*_e_, and then resulted in the poor growth performances (as *WG*_w_ and *SGR*_w_) from birth to first-time reproduction in the present study. However, the highest *RL*_e_ value of 119% was observed in the H ration group in the juvenile stage of *G. affinis*. This is much higher than that reported in many other species of fish such as *Acipenser schrenckii*, *Esox lucius*, and *Mystus vittatu* [23,24,25]. In addition, the *FCE*_e_ in the juvenile stage was also higher than that reported in *Phoxinus phoxinus* and *Gobiocypris rarus* [26,27]. It suggested that the *G. affinis* likely have the greater ability to assimilate enough energy from food for the somatic and gonad growth when supplied with enough food, which maintained the rapid growth and reproduction of *G. affinis*.

On the contrary, the marked increase of *FCE*_e_ was observed in the L and M ration levels from birth to first-time sexual maturity, which inferred that *G. affinis* have likely improved digestibility that afforded it more energy from limited food. This physiological adaptation has contributed to the compensatory growth of fish, which encounter the dietary restriction [28,29,30], and it was also beneficial to the reproduction of fish under such circumstances. As a result, the female *G. affinis* in the present study exhibited growth with high *RL*_e_ and *FCE*_e_ values in the early juvenile stage, which is consistent with the previous reports [31]. This strategy of rapid growth is also beneficial to the predator’s avoidance and provision of sufficient energy for gonad development and vitellogenesis occurrences, which require much more energy accumulation.

The *RL*_e_ in the developmental stage of 17 days of age to first-time spawning was higher than that in “0–16 days of age” stage. However, the *SGR*_w_ and *FCE*_e_ were all decreased. It is possible that more energy was allocated to the oocyte and embryonic development, and thus reducing the energy available for the somatic growth. This could likely be the cause of the decrease for *FCE*_e_ in the L and M groups in the stage of “First-time sexual maturity—First-time spawning”. The values of *CF* in each ration level for the “17 days of age—First-time sexual maturity” stage were higher than those in the other stages, which potentially provided good conditions for the female *G. affinis* to successfully breed and reproduce.

Food intake is critical to the amount of energy available for investment in growth and reproduction processes in fish [31]. A greater proportion of energy is invested in growth prior to sexual maturity [2,32]. Although the absolute energy allocated to somatic and gonad growth were decreased with decreasing ration levels in the “17 days of age—First-time sexual maturity” stage, however, the percentage of energy allocated to them were all increased markedly. It suggested that a higher percentage of energy was invested in both somatic and gonad growth to achieve sexual maturity of female *G. affinis* in the case of food restriction. Similarly, the absolute energy allocated to somatic and gonad growth were decreased with decreasing ration levels when the female *G. affinis* entered into the “First-time sexual maturity—First-time spawning” stage, however, the percentage of energy allocated to gonad growth was less affected by ration levels. This suggests that energy was likely preferentially used for embryo development in the case of food restriction, which was useful to the reproduction of female *G. affinis* in that adverse environment.

The ovary mass and number of oocytes/embryos were all markedly increased with the increasing ration levels in the stages of “17 days age—First sexual maturity” and “First sexual maturity—First production”, which is consistent with previous findings in other teleost fish species [33,34]. It indicated that *G. affinis* can produce more offspring when there is enough food. Lower ovary weight and oocytes/embryos number existed in L and M groups; however, the values of *GSI* and oocytes/embryos weight have not changed significantly. It suggested that the quality of the embryos in food restriction groups should be as good as those in satiation group. Reznick (1981) reported that offspring size was determined genetically, but not by ration level during pregnancy [35]. The size of eggs or 0 days old larvae of hybrid tilapia were also unaffected by the ration levels [7]. The larvae’s body size of the *G. affinis* could affect their survival, growth rate and competitive ability [35,36]. However, the offspring, which their female parents were supplied with different ration levels, also showed similar growth performances in the present study, proving that the ration levels have no effect on embryo development and offspring growth.

In the present study, the first-time sexual maturity at 52 days was observed in the H group, and this was consistent with the previous result reported by Vondracek *et al.* (1988) [22]. However, the time taken to reach sexual maturity in the M and L groups were 55 and 58 days, respectively, suggesting that the sexual maturity was delayed by food restriction in the present study. Nevertheless, the *G. affinis* can produce several generations every year [10,18,37], and be inclined to produce many offspring in case of plentiful food supply. However, the reproduction season of *G. affinis* varied due to the climatic differences where it inhabited. The reproduction season ranged from March to November in the Guangzhou city, China [38], suggesting that *G. affinis* could produce offspring during the majority of the year. Furthermore, the sexual maturity and spawning time of *G. affinis* were shorter compared with *Tanichthys albonubes*, which was a small native fish species in Guangdong province, China. It reported that about 72-days post-hatch of *T. albonubes* reached sexual maturity and the *G. affinis* could prey on their larvae in the wild [34,39], which was also one of the greatest threats of *T. albonubes* extinction. All these enhanced the ability of *G. affinis* to survive and adapt to adverse environmental conditions.

## 4. Experimental Section

### 4.1. Experimental Fish

Large numbers of adult female *G. affinis* were collected with a net from a ditch associated with fish cultivating ponds near Guangzhou city, China. Subjects were kept in an aquarium until they appeared to be close to parturition and, then each subject was individually housed during reproduction. A total of 1600 *G. affinis* larvae of 0 days old produced on the same day were chosen randomly for the present study.

### 4.2. Experimental Design and Management

Each selected larva was individually grown in a 500 mL glass beaker containing 400 mL of fresh water. Fish subjects were randomly divided into three feeding groups for the experimental treatment. High ration level (H) represents satiation feeding; Medium ration level (M) was 50% of the high ration level and Low ration level (L) was 25% of the high ration level. The food amount of high ration was determined by preliminary testing, where excess food was supplied and uneaten food was collected an hour later. The food intake was calculated by the weight of supplied and surplus food for fish. Half of the water in each glass beaker was replaced daily. Uneaten food and feces were collected by net with 80 mesh from the glass beakers in the morning before feeding. Collected surplus food from each beaker was separately oven-dried at 60 °C and weighed, and then stored at −20 °C for chemical analysis and energy determination. Throughout the experimental period, standard conditions were maintained at a water temperature of 28 ± 0.5 °C using a water bath which the *G. affinis* can well growth and reproduction between 20 and 34 °C [40]. The photoperiod was 16L:8D in the present study. Dissolved oxygen (DO), pH, ammonia and total nitrogen (TN) were monitored weekly.

The fish was divided into four developmental stages throughout the experimental period: (1) 0–16 days of age: the first-generation offspring from birth to 16 days old represent early developmental stage of first-generation juveniles, because the anal fin was modified into the copulatory organ at around the 16th day; (2) 17 days of age—First-time sexual maturity: represents the first developmental stage of gonad, which spanning from the 17th day to the time of oocytes attains maturity; (3) First-time sexual maturity—First-time spawning: represents the first gestational stage, which spanning from mating to spawning; and (4) 0–11 days of age of second-generation offspring: represents early developmental stage of second-generation juveniles, because the anal fin began to metamorphose visibly at around the 11th day and we can determine gender by it.

From 0 to 16 days old, each fish was fed with 24 h post-hatched live *Artemiasalina* nauplii three times each day at 09:00, 17:00, and 23:00. From 17 days old to the first-time spawning, the fish were fed with frozen *Chironomus* sp. twice daily at 09:00 and 21:00. Also a supplementary ration of 24 h post-hatched live *A. salina* nauplii at 500 ind. d^−1^ (the number of individuals each deciliter) was given per fish at 17:00. All *Chironomus* sp. were rinsed and dried en masse on absorbent gauze and weighed prior to feeding. Preliminary testing indicated that *G. affinis* readily accepted *A. salina* nauplii as a nutritional supplement. Both the frozen *Chironomus* sp. (Dolphin fish feed Co., Ltd., Guangzhou, China) and *A. salina* eggs (Bonasse biochemical feed Co., Ltd., Zhanjiang, China) were bought from the same batch. *A. salina* eggs were hatched in the laboratory under similar environmental conditions.

At the start of the experiment, 20 first-generation fish subjects (0 days old) were selected randomly for body weight measurement to the nearest 0.01 mg and standard length measurement to the nearest 0.01 mm. At 10–15 days old, gender was determined according to juvenile anal fin metamorphosis, and all males were removed. At 16 days old, 40 female juveniles from each treatment group were randomly selected and killed with an overdose of anesthetic (MS-222) for body weight and standard length measurements.

The remaining juvenile females of 16 days old were reared to first-time sexual maturity. A total of 60 females at first-time sexual maturity were randomly selected for weight and length measurement, while 180 females in each ration group were randomly selected and paired with the adult male *G. affinis* for breeding in separate aquaria for 24 h. A male:female ratio of 1:2 was used during breeding, and no food was provided during this period. Female fish of each ration group were collected after breeding, and cultured individually in 500 mL glass beakers. The ages at which the first 20 females of each ration group produced offspring were recorded. When 90% of the remaining females at the same ration level appeared to near parturition, all gravid females were sampled. A total of 20 samples of 0 days old offspring in each ration group were randomly selected for body weight and length measurement from the first 20 female fish that produce offspring.

In order to assess the health status of the offspring, 30 second-generation offspring (0 days old) from each ration group were individually housed in 500 mL glass beakers and fed on the *A. salina* nauplii by satiation three times daily. Out of these, 10 fish were randomly sampled from each group for the measurement of body weight and length when the anal fin began to metamorphose visibly. With the exception of the 0 days old group, all fish were provided with no rations for 24 h prior to the measurement of body weight.

### 4.3. Measurement and Calculation

Each fish was weighed and measured after surface water was removed by blotting with absorbent gauze. Subjects were sacrificed at first-time reproduction by overdose of anesthetic (MS-222), and ovaries were excised and weighed individually. Then the oocytes of each ovary were isolated for counting and weighing. In addition, the development of ova and embryos were counted and weighed in these samples. All samples were dried to constant weight at 60°C and weighed, and then stored at −20°C for energy determination. Energy content was measured by C5000 oxygen bomb calorimeter (IKA, Staufen, Germany). Each sample analysis was conducted at least twice.

Ration levels in terms of energy (*RL*_e_, % body energy content day^−1^) was calculated as follow:

$$RL_{\text{e}}=\frac{C_{\text{e}}}{\frac{\left(E_{\text{t}}+E_{\text{i}}\right)}{2}\times t}\times 100$$

where *C*_e_ was the total food energy consumption (kJ) in the developmental stage, which was calculated by multiplying total food intake (mg) with food energy content (kJ·mg^−1^); *E*_t_ was the body energy content (kJ) of fish at the end of each developmental stage, which was calculated by multiplying final body weight (mg) with the final energy content (kJ·mg^−1^) of fish; *E*_i_ was the body energy content in fish at the start of each developmental stage and, it was calculated by multiplying the initial body weight with the initial energy content of fish; *t* was the days contained in each developmental stage.

The condition factor (*CF*), gonad-somatic index (*GSI*, %), specific growth rates of wet weight (*SGR*_w_, %·day^−1^), wet weight gained each day in fish (*WG*_w_, mg·day^−1^), and food conversion efficiency in terms of energy (*FCE*_e_, %) were calculated as follows:

$$CF=\frac{W_{\text{t}}}{L^{3}}\times 100$$

$$GSI=\frac{W_{\text{g}}}{W_{\text{t}}}\times 100$$

$$SGR_{\text{w}}=\frac{lnW_{\text{t}}-lnW_{\text{i}}}{t}\times 100$$

$$WG_{\text{w}}=\frac{W_{\text{t}}-W_{\text{i}}}{t}\times 100$$

$$FCE_{\text{e}}=\frac{E_{\text{t}}-E_{\text{i}}}{C_{\text{e}}}\times 100$$

where *W*_t_ and *W*_i_ were the final and initial body weight (wet weight, mg) at each developmental stage, respectively; *L* was the body length (mm) and *W*_g_ was the gonad weight (mg) of fish in each developmental stage.

### 4.4. Statistical Analysis

Statistical analysis of the data was subjected to one-way ANOVA and LSD multiple range analysis using SPSS 15.0 (SPSS Inc., Chicago, IL, USA). Results were presented as means ± SD and differences were considered statistically significant at *p* < 0.05.

## 5. Conclusions

In summary, the current study indicates that *G. affinis* can adapt its somatic growth and gonad development to food restriction by the adjustment of energy distribution in different developmental stages. Also, the female *G. affinis* can produce a quantity of healthy offspring with limited food supply by increasing the energy allocated to gonad development, reducing fecundity and delaying spawning time. These life strategies ensured that at least some viable offspring are produced in adverse environments, including those with low food resource availability. As a result, *G. affinis* can survive and thrive in a much wider range of environmental conditions than many other fish species, exhibiting a characteristic specific to invasive fish species.
